# Androgen regulation of the androgen receptor coregulators

**DOI:** 10.1186/1471-2407-8-219

**Published:** 2008-08-01

**Authors:** Alfonso Urbanucci, Kati K Waltering, Hanna E Suikki, Merja A Helenius, Tapio Visakorpi

**Affiliations:** 1 Institute of Medical Technology, University of Tampere and Tampere University Hospital, FI-33014 University of Tampere, Tampere, Finland

## Abstract

**Background:**

The critical role of the androgen receptor (AR) in the development of prostate cancer is well recognized. The transcriptional activity of AR is partly regulated by coregulatory proteins. It has been suggested that these coregulators could also be important in the progression of prostate cancer. The aim of this study was to identify coregulators whose expression is regulated by either the androgens and/or by the expression level of AR.

**Methods:**

We used empty vector and AR cDNA-transfected LNCaP cells (LNCaP-pcDNA3.1, and LNCaP-ARhi, respectively), and grew them for 4 and 24 hours in the presence of dihydrotestosterone (DHT) at various concentrations. The expression of 25 AR coregulators (*SRC1*, *TIF2*, *PIAS1*, *PIASx*, *ARIP4*, *BRCA1*, *β-catenin*, *AIB3*, *AIB1*, *CBP*, *STAT1*, *NCoR1*, *AES*, *cyclin D1*, *p300*, *ARA24*, *LSD1*, *BAG1L*, *gelsolin*, *prohibitin*, *JMJD2C*, *JMJD1A*, *MAK*, *PAK6 *and *MAGE11*) was then measured by using real-time quantitative RT-PCR (Q-RT-PCR).

**Results:**

Five of the coregulators (*AIB1*, *CBP*, *MAK*, *BRCA1 *and *β-catenin*) showed more than 2-fold induction and 5 others (*cyclin D1*, *gelsolin*, *prohibitin*, *JMJD1A*, and *JMJD2C*) less than 2-fold induction. Overexpression of AR did not affect the expression of the coregulators alone. However, overexpression of AR enhanced the DHT-stimulated expression of *MAK*, *BRCA1*, *AIB1 *and *CBP *and reduced the level of expression of *β-catenin*, *cyclinD1 *and *gelsolin*.

**Conclusion:**

In conclusion, we identified 5 coactivators whose expression was induced by androgens suggesting that they could potentiate AR signaling. Overexpression of AR seems to sensitize cells for low levels of androgens.

## Background

Prostate cancer is the most and second most common male malignancy in the USA, and Europe, respectively [1,2]. The androgen-dependence of the growth of prostate cancer has been known for a long time [3]. The importance of the androgens was recently, once again, demonstrated in a large randomized study. In the study the use of finasteride, an inhibitor of 5α-reductase, which converts testosterone into more potent 5α-dihydrotestosterone, was used for prevention of prostate cancer [4]. Almost 25% reduction in the prevalence of prostate cancer was observed in the treatment compared to a placebo arm. Due to the androgen dependence, the golden standard treatment for advanced prostate cancer has been androgen withdrawal (i.e. castration) for the last half century [5]. The treatment is palliative and although most patients respond to it, the disease will eventually progress [6]. Such tumors, emerging during the androgen withdrawal, are called hormone-refractory ones.

The molecular mechanisms by which prostate cancer cells become resistant to endocrine therapy remain incompletely known. However, the key role of androgens and androgen receptor (AR), not just in early development but also in the progression of prostate cancer has now been demonstrated [7]. The recent finding on genetic rearrangement leading to formation of *TMPRSS2:ERG *fusion gene provides a model for molecular mechanisms how androgens act in promoting the early development of prostate cancer. Due to the rearrangement, the putative oncogene *ERG *becomes androgen regulated [8,9]. It has now also become apparent that the emergence of hormone-refractory prostate cancer is due to reactivation of AR-mediated signaling. It has been experimentally shown that overexpression on AR is required and it is sufficient to transform the growth of prostate cancer cells from androgen-dependence to -independence [10]. Furthermore, it has been shown that hormone-refractory tumors overexpress AR, and one-third of them contains amplification of the *AR *gene [11]. Mutations in the coding region of *AR *have also been found in antiandrogen treated prostate cancers [12,13]. And at least some of the mutations alter the sensitivity of the receptor to other steroid hormones or anti-androgens, such as 17 β-estradiol (E2) or hydroxyflutamide (HF) [14,15].

The transactivation of AR involves several coregulatory proteins [16,17]. Large number of coregulatory proteins have already been identified [18-20]. It has been suggested that changes in the expression of the coregulators may be involved in the development and progression of prostate cancer [18,21,22]. Since androgens and AR are known to be important in the prostate cancer tumorigenesis, it is possible that they also regulate the expression of the coregulators.

In order to identify AR coregulators whose expression is regulated by androgens, we measured the expression of 20 putative coactivators: *SRC1 *(alias *NCOA1 *nuclear receptor coactivator 1), *TIF2 *(alias *NCOA2 *nuclear receptor coactivator 2), *PIAS1 *(protein inhibitor of activated *STAT, 1*), *PIASx *(alias *PIAS2 *protein inhibitor of activated STAT, 2), *ARIP4 *(alias *RAD54L2 RAD54*-like 2 (*S. cerevisiae*)), *BRCA1 *(breast cancer 1, early onset), *β-catenin *(alias *CTNNB1 *catenin (cadherin-associated protein), beta 1, 88 kDa), *AIB3 *(alias *NCOA6 *nuclear receptor coactivator 6), *AIB1 *(alias *NCOA3 *nuclear receptor coactivator 3), *CBP *(alias *CREBBP CREB *binding protein (Rubinstein-Taybi syndrome)), *STAT1 *(signal transducer and activator of transcription 1, 91 kDa), *p300 *(alias *EP300 *E1A binding protein p300), *ARA24 *(alias *RAN*, member *RAS *oncogene family), *LSD1 *(lysine-specific demethylase 1, alias *AOF2 *amine oxidase (flavin containing) domain 2), *BAG1L *(*BCL2*-associated athanogene, isoform 1L), *gelsolin *(*GSN *(amyloidosis, Finnish type)), *JMJD2C *(alias JHDM3C/GASC1 jumonji domain containing 2C), *JMJD1A *(alias JHMD2A jumonji domain containing 1A), *MAK *(male germ cell-associated kinase), *MAGE11 *(alias *MAGEA11 *melanoma antigen family A, 11) and 5 putative corepressors: *NCoR1 *(nuclear receptor co-repressor 1), *AES *(amino-terminal enhancer of split), *cyclin D1 *(alias *CCND1*), *prohibitin *(*PHB*) and *PAK6 *(*p21*(*CDKN1A*)-activated kinase 6) by using quantitative reverse transcription-PCR (Q-RT-PCR) in AR positive prostate cancer cell line LNCaP treated with different concentrations of DHT. To mimic the common overexpression of AR in hormone-refractory prostate cancer, we also stable transfected AR to LNCaP cell line. The LNCaP-ARhi cells express about 10-folds more AR mRNA and 3 to 4-folds more AR protein than that of the control (empty vector-transfected cell line: LNCaP-pcDNA3.1). The cell model was used to study whether the level of AR has an effect on the mRNA expression of the coregulators.

## Methods

### Cell culture protocols and transfections

**Figure 1:**
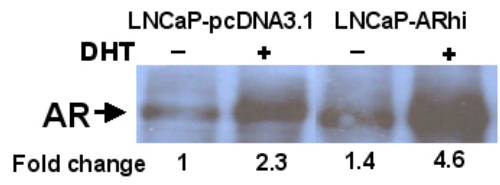
Western blot analysis of AR in empty vector transfected (LNCaP-pcDNA3.1) and AR-cDNA transfected (LNCaP-ARhi) LNCaP cells. Cells were grown either in the absence or presence of DHT. Nuclear fraction of LNCaP-ARhi shows clearly higher AR expression than the control LNCaP-pcDNA3.1. The quantification of the bands by ImageJ is given below the bands.

pcDNA3.1(+) empty expression vector (Invitrogen Inc., Carlsbad, CA, USA) and pcDNA3.1(+) inserted with AR coding region were stable transfected into LNCaP (ATCC, Manassas, VA, USA) with Lipofectamine-Plus transfection reagent (Invitrogen Inc.). Transfected clones were selected with 400 μg/ml geneticin (G418) for 2 weeks. The mRNA level of AR was examined from purified clones using Northern blotting and Q-RT-PCR with Light-Cycler (Roche Inc., Mannheim, Germany). The protein level of AR was analyzed using Western blotting with anti-AR antibody (441, NeoMarkers-Lab Vision Corporation, Fremont, CA, USA). LNCaP-pcDNA3.1 (transfected with empty pcDNA3.1-vector as a control) and LNCaP-ARhi (transfected with AR cloned into pcDNA3.1+ vector) cell lines were cultured according to the ATCC protocol with the addition of Geneticin 200 μg/ml (Invitrogen Inc.). LNCaP-ARhi has about 10 times higher mRNA level of AR and 3 to 4 times higher protein level of AR than that of the LNCaP-pcDNA3.1 (Figure 1).

LNCaP-pcDNA3.1 and LNCaP-ARhi cells in the end of the exponential growing phase were divided in 1:4 ratio plates in RPMI1640 phenol free medium with 5% charcoal/dextran-treated (CCS) FBS (Hyclone Inc., South Logan, UT, USA), 1% glutamine (Invitrogen Inc.) and 1% penicillin-streptomycin (BioWhittaker Inc., Walkersville, MD, USA) for 4 days. The medium was then changed to phenol free RPMI1640 including 10% CCS-FBS (Hyclone Inc.), 1% Glutamine (Invitrogen Inc.), 1% Pest (Invitrogen Inc.) and 0, 0.1, 1.0, 10 or 100 nM DHT. The cells were harvested into TRIZOL reagent (Life Technologies Inc., Gaithersburg, MD, USA) after 4 h and 24 h, followed by total RNA isolation according to the manufactures protocol.

### RT-PCR

The cDNA was synthesized with AMV Reverse Transcriptase and oligo(dT)12–18 primer according to the manufacturer's protocol (Finnzymes, Espoo Finland). The standards were prepared mixing total RNA from untreated LNCaP cells and universal RNA (Clontech Laboratories, Inc., Mountain View, CA, USA) in a ratio of 1:5. After first strand cDNA synthesis, serial dilutions corresponding to 1000, 200, 40, 8, 1.6, 0.32, 0.064 μg of RNA pool were prepared and stored in aliquots. The PCR reactions were performed in LightCycler apparatus (Roche Inc.) using an LC Fast Start DNA SYBR Green I Kit (Roche Diagnostics, Mannheim, Germany). The final volume of each reaction was 20 μl containing 2 μl of cDNA sample (or standard), 4 mM MgCl_2 _(except for *PAK6 *(5 mM) and *JMJD1A *(3 mM)), 0.5 μM each primer, and 1× ready-to-use SYBR Green I reaction mix including Taq DNA polymerase, reaction buffer and a deoxynucleotide triphosphate mix. The cycling conditions were designed according to the manufacturer's guidelines which are given in additional file 1: primers' table. The primers were designed for amplifying regions of the mRNAs derived from different exons to avoid amplification of genomic DNA. The melting curve analysis with the LightCycler together with 1.5% agarose gel electrophoresis of the products were used to ensure that right size product without significant background was amplified in the reaction. The expression levels of the coregulators were normalized to the expression levels of TATA-box binding protein (*TBP*) as previously described [23].

### Western analysis

Cytoplasmatic and nuclear proteins were isolated from subconfluent cells of LNCaP-ARhi. Proteins were separated in 10% SDS-PAGE gel followed by transfer to a membrane (Immobilon-P, Millipore Corp., Billerica, MA) using BIORAD transblot^® ^semi-dry transfer cell (Bio-Rad Laboratories, Inc). After blocking in PBS 0.1% Tween 5% BSA (or 5% non-fat dry milk), the membranes were incubated with primary antibody (AR: dilution 1: 200 Androgen Receptor Ab-1 (AR441) Neomarkers, Fremont, CA; β-catenin: dilution 1:2000 BD Transduction Laboratories, Inc.; MAK: dilution 1:500 MAK antibody (C-term), ABGENT San Diego, CA; CBP: dilution 1:1000, R&D Systems, Inc.) over night at +4°C. After washes and incubation with secondary antibody (DAKO A/S, Denmark) the bound antibody was visualized on autogradiography film using Western Blotting Luminol Reagent (Santa Cruz, Inc.) according to the manufacturer's protocol. Pan-actin antibody (dilution 1:1000 pan AB-5, clone ACTN05, Neomarkers, Fremont, CA;) was used as a reference.

### Statistical analysis

Grubb's test was used to detect the outlier values in the repetitive runs for each gene. One-way ANOVA (Parametric) test with Bonferroni post-test was used to evaluate the statistical significance of the expression level's changes.

## Results

**Figure 2:**
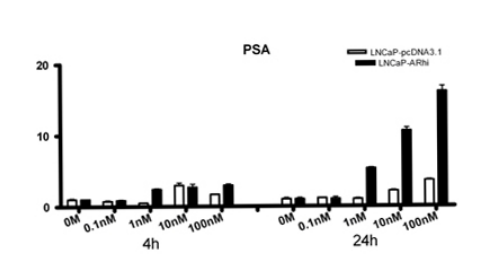
Expression of PSA in LNCaP-pcDNA3.1 and LNCaP-ARhi according to Q-RT-PCR. The cells were cultured in presence of DHT at different concentrations. After 4 and 24 hours, the cells were collected and expression of *PSA *and *TBP *mRNA was measured in triplicates by Q-RT-PCR. The bars and whiskers represent mean + S.E.M. of *PSA*/*TBP *values normalized against the 0 M of each time point.

To identify androgen regulated coregulators, empty vector transfected LNCaP (LNCaP-pcDNA3.1) was grown in different concentrations of DHT. The total RNA was then collected at two time points (4 h and 24 h). To confirm the success of the DHT stimulation, the expression of prostate-specific antigen (*PSA*), known androgen regulated gene was first measured (Figure 2). *PSA *was strongly induced by DHT (p < 0.0001 at 4 h and 24 h).

Next, we screened expression of *SRC1*, *TIF2*, *PIAS1*, *PIASx*, *ARIP4*, *BRCA1*, *β-catenin*, *AIB3*, *AIB1*, *CBP*, *STAT1*, *NCoR1*, *AES*, *cyclin D1*, *p300*, *ARA24*, *LSD1 *and *BAG1L *coregulators in triplicates (three independent Q-RT-PCRs' runs). Of the coregulators, *PIASx *(p = 0.0601 at 4 h and p = 0.6488 at 24 h), *BRCA1 *(p = 0.4172 at 4 h and p = 0.0377 at 24 h), *β-catenin *(p = 0.0272 at 4 h and p = 0.4158 at 24 h), *AIB3 *(p = 0.0690 at 4 h and p = 0.1455 at 24 h), *AIB1 *(p = 0.0401 at 4 h and p = 0.2541 at 24 h), *CBP *(p = 0.0184 at 4 h and p = 0.0588 at 24 h), *cyclin D1 *(p = 0.2039 at 4 h and p = 0.4432 at 24 h), *ARA24 *(p = 0.0220 at 4 h and p = 0.2502 at 24 h) and *BAG1L *(p = 0.0359 at 4 h and p = 0.3981 at 24 h), showed dose dependent response to androgens, whereas *SRC1*, *TIF2*, *PIAS1*, *ARIP4*, *STAT1*, *NCoR1*, *AES*, *p300 *and *LSD1 *did not.

**Figure 3:**
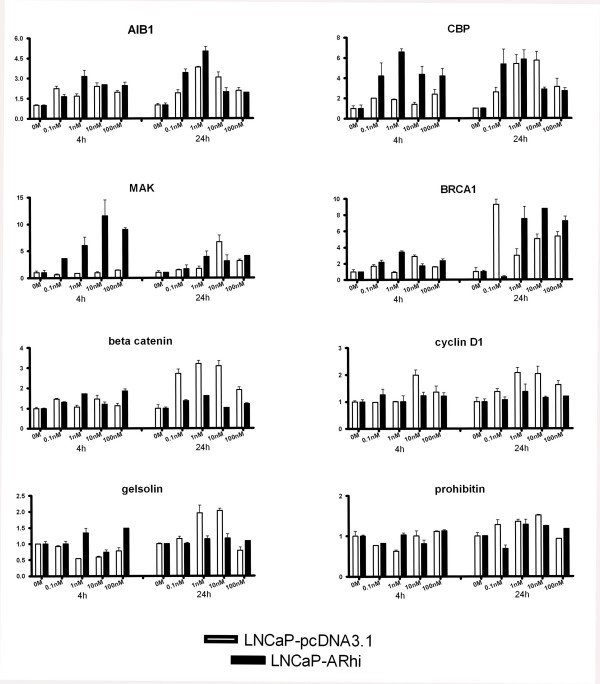
The expression of *AIB1*, *CBP*, *MAK*, *BRCA1*, *β-catenin*, *cyclin D1*, *gelsolin *and *prohibitin *in LNCaP-pcDNA3.1 and LNCaP-ARhi at 4 and 24 hours according to Q-RT-PCR. The measurements were done in triplicates. The bars and whiskers represent mean + S.E.M. of each gene against the TBP, normalized against the 0 M of each time point. p-values are given in Table 1.

**Figure 4:**
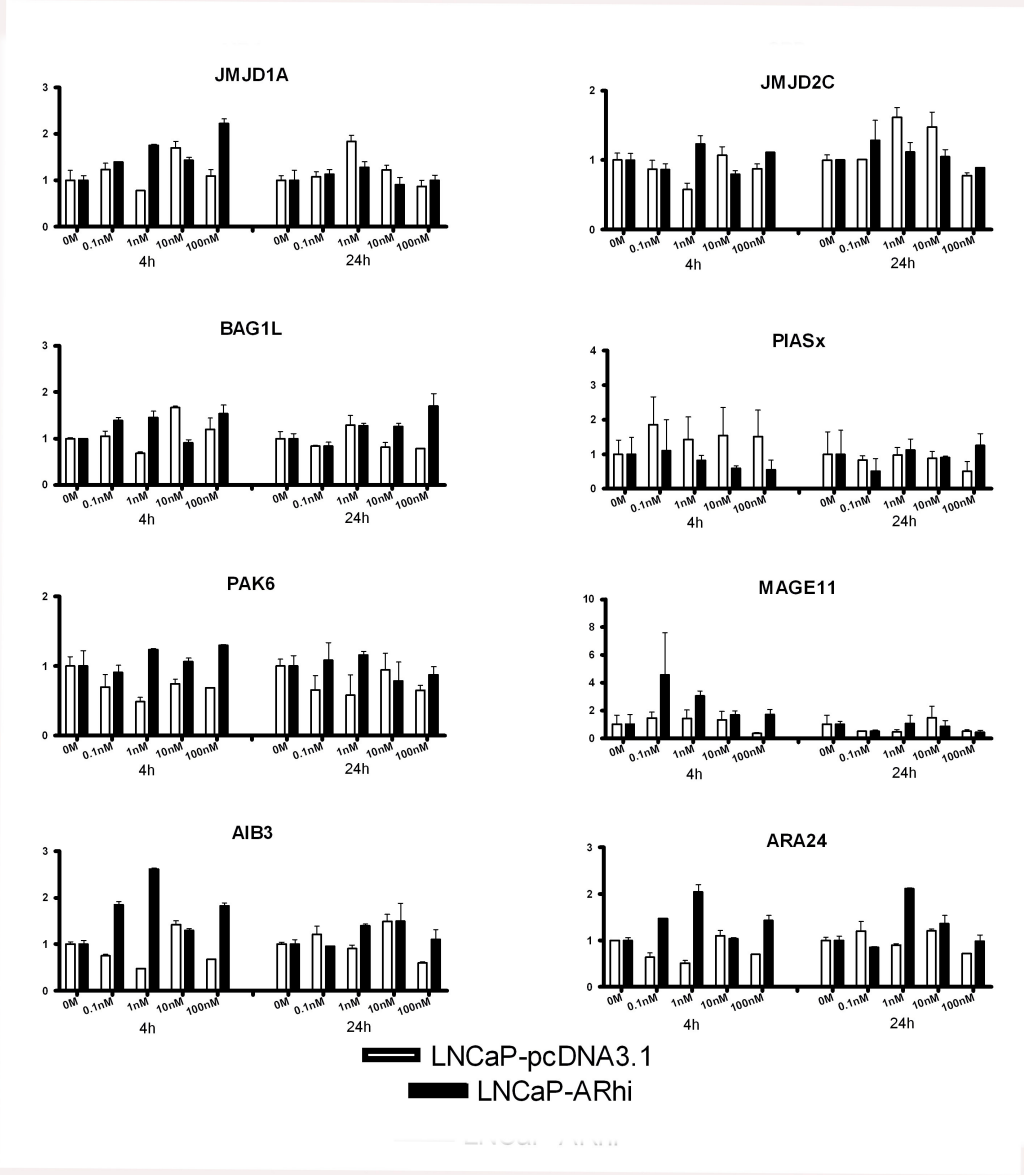
The expression of *JMJD1A*, *JMJD2C*, *BAG1L*, *PIASx*, *PAK6*, *MAGE11*, *AIB3*, and *ARA24 *in LNCaP-pcDNA3.1 and LNCaP-ARhi at 4 and 24 hours according to Q-RT-PCR. The measurements were done in triplicates. The bars and whiskers represent mean + S.E.M. of each gene against the TBP, normalized against the 0 M of each time point. p-values are given in Table 1.

*PIASx*, *BRCA1*, *β-catenin*, *AIB3*, *AIB1*, *CBP*, *cyclin D1*, *ARA24 *and *BAG1L *were selected for additional Q-RT-PCR runs in triplicates. In addition, expression of *gelsolin*, *prohibitin*, *JMJD2C*, *JMJD1A*, *MAK*, *PAK6 *and *MAGE11 *was measured in triplicates. Figures 3 and 4 show the expression pattern of these 16 coregulators upon DHT stimulation. The genes that showed significant dose dependent expression were *AIB1*, *CBP*, *MAK*, *BRCA1*, *β-catenin*, *cyclin D1*, *gelsolin*, *prohibitin*, *JMJD1A*, and *JMJD2C*. Table 1 indicates the p-values of the sixteen genes. *Gelsolin *showed reduced and increased expression at 4 and 24 hour time points, respectively. Others exhibited an androgen-dependent increase in the expression of the mRNAs. However, only *AIB1*, *CBP*, *MAK*, *BRCA1*, and *β-catenin *showed more than 2-folds induction after stimulation with DHT.

**Table 1:** p-values of the One-way ANOVA (Parametric) test

Gene	LNCaP-pcDNA3.1		LNCaP-ARhi	
	
	4 h	24 h	4 h	24 h
*AIB1*	0.0019	< 0.0001	0.0023	< 0.0001
*CBP*	0.0478	0.0103	0.0035	0.0205
*MAK*	0.1385	0.0014	0.0090	0.1625
*BRCA1*	0.0007	< 0.0001	0.0022	< 0.0001
*B-catenin*	0.0336	< 0.0001	< 0.0001	< 0.0001
*Cyclin D1*	0.0094	0.0094	0.6382	0.4697
*Gelsolin*	0.0016	< 0.0001	0.0029	0.4211
*Prohibitin*	0.0291	0.0084	0.0114	0.0053
*JMJD1A*	0.0276	0.0009	< 0.0001	0.4592
*JMJD2C*	0.0588	0.0064	0.0259	0.6450
*BAG-1L*	0.0029	0.1467	0.0244	0.0150
*PIASx*	0.9371	0.8551	0.8224	0.7616
*PAK6*	0.1075	0.4876	0.3359	0.6410
*MAGE11*	0.7256	0.5952	0.6438	0.6014
*AIB3*	< 0.0001	0.0135	< 0.0001	0.3818
*ARA24*	0.0016	0.0276	0.0007	0.0013

p-values of the 16 genes were measured in triplicate in the second round analysis.

In order to evaluate the effect of the level of AR expression on the expression of the coregulators, we utilized LNCaP-based overexpression model (LNCaP-ARhi). The *PSA *response in the LNCaP-ARhi cell line at 24 hours was significantly (p < 0.0001) stronger than in the empty vector transfected cell line (LNCaP-pcDNA3.1) (Figure 2). PSA expression was stimulated already in 1 nM concentration in the LNCaP-ARhi, whereas the stimulation was evident in 10 nM in the LNCaP-pcDNA3.1.

None of the coregulators showed significant alterations in the expression in 0 M DHT between the LNCaP-pcDNA3.1 and LNCaP-ARhi. However, induction of expression in lower concentrations of DHT in LNCaP-ARhi compared to LNCaP-pcDNA3.1 was found in *MAK*, *CBP*, *AIB1*, and *BRCA1*. In contrast, the androgen regulation of *β-catenin*, *cyclin D1 *and *gelsolin*, which was apparent in LNCaP-pcDNA3.1 at 24 h was less strong in LNCaP-ARhi (Figures 3 and 4).

Western blot analyses of β-catenin, CBP and MAK were in concordance with the Q-RT-PCR results (see additional file 2: WB analysis).

## Discussion

We systematically studied the androgen regulation of the expression of twenty-five AR coregulators. 5 (20%) coregultators showed statistically significant over 2-fold induction by DHT. These were *AIB1*, *CBP*, *MAK*, *BRCA1 *and *β-catenin*. In addition, expression of five (20%) other coregulators (*cyclin D1*, *gelsolin*, *prohibitin*, *JMJD1A*, and *JMJD2C*) was significantly, but less than 2-fold, induced by androgens.

*AIB1 *(Amplified in Breast Cancer 1) encodes for a well characterized protein with histone acetyltransferase activity. It is thought to be a nuclear receptor coactivator that interacts with various nuclear hormone receptors enhancing their transcriptional activity in a hormone dependent fashion. It is a member of the p160/steroid receptor coactivator (SRC) family and recruits p300/CBP-associated factor, with histone acetylation activity, and CBP (CREB binding protein) as a part of the transcription multisubunit coactivation complex [19,24]. *AIB1 *has been found to be amplified in about 10% of breast cancers [25]. Previous studies have indicated that estrogens might have a suppressive role in respect to the expression of AIB1 [26,27]. Here we found that at 4 hours time point *AIB1 *is nearly 2-fold induced by any concentration of DHT while at 24 hours time point a 4-fold induction was observed. The highest induction was at 1 nM DHT while at 10 nM the expression was declined. We used web-based TESS (Transcription Element Search Software) program and TRANSFAC database [28] to search for canonical binding sites for AR in the 2 kb upstream the transcription start site (TSS) of *AIB1*. The program identified several glucocorticoid receptor (GR) responsive elements (GREs) and two putative androgen responsive elements (AREs) at -527 and at -1291 bp from the TSS.

Similarly to *AIB1*, *CBP *was nearly 2-folds induced at 4 hours time point by any concentration of DHT while at 24 hours time point a 4-folds induction can be observed, although such induction can be seen also in 10 nM DHT concentrated media. In contrast with our results, Comuzzi et al. have suggested that *CBP *expression is down-regulated after treating LNCaP cells with synthetic androgen methyltrienolone (R1881) for 48 hours at concentrations of 0.01 nM and 1 nM [29].

*MAK *(Male Germ Cell-Associated Kinase) is a recently identified AR coactivator [30] that initially has been found to be strongly androgen-regulated in mice kidney [31]. In addition, it has previously been demonstrated that *MAK *is transcriptionally induced by DHT in LNCaP cells showing 9-fold induction by 10 nM DHT at 24 hours [32]. This is confirmed by our finding of 6.5-fold induction at 24 hours time point by 10 nM DHT. MAK has been found to be associated with AR and being corecruited in the transcriptional complex enhancing AR activity. Furthermore the modulation of its expression strongly alters the vitality of the cells affecting AR pathway mediated signaling [30]. Jia et al. have shown that *MAK *promoter is a direct target of AR and identified putative ARE sites within the promoter of *MAK *(about -3500 bp from the TSS), and that AR is recruited to the *MAK *promoter after DHT stimulation according to chromatin immunoprecipitation (ChIP) analysis [33]. Altogether the data indicates that the expression of *MAK *is regulated by androgens and since it functions as an AR co-activator, it forms a putative feedback loop augmenting the effects of androgens.

*BRCA1 *(breast cancer 1) is a well characterized gene in breast cancer since mutations in this gene are responsible of at least 40% of inherited breast cancers and more than 80% of inherited ovarian cancers [34,35]. BRCA1 is also an AR coactivator that has been demonstrated to enhance AR transactivation in prostate and breast cancer cell lines with a synergistic effect with other p160 coactivators [36]. Our data shows an androgen-mediated induction of the expression of *BRCA1*. It has previously been demonstrated that *BRCA1 *is an estrogen induced gene in breast and ovarian cancer cells [37,38].

β-catenin is an adherens junction protein with altered expression in various tumor types. Its fundamental role as a multifunctional oncoprotein has been established [39]. The gene encodes for a protein that interacts with AR in close proximity with p160 coactivator family and it is thought to be important for the formation of the AR transcription complex [40,41]. There is some evidence that AR could also have an important role as a scaffolding protein in β-catenin translocation from the cytoplasm into the nucleus [42-44]. β-catenin has been shown to augment AR transcriptional function in a ligand-dependent fashion [43,45]. Here we show that *β-catenin *is induced up to 2.5-folds at 24 hours time point, already at a concentration of 0.1 nM of DHT. The induction reaches then the 3-folds and declines at 2-folds at 100 nM showing a significant androgen regulation.

In addition to the above mentioned coregulators, *cyclin D1*, *gelsolin*, *prohibitin*, *JMJD1A *and *JMJD2C *showed a significant dose dependent effect in their expression level mainly at 24 hours time point. However, the induction was very mild, less than 2-fold, suggesting either only weak androgen effect or secondary mechanisms for the induction.

It has been demonstrated that AR is commonly overexpressed in hormone-refractory prostate cancer [11,46,47]. In addition, it has been shown that the overexpression is capable to transform androgen dependent growth of prostate cancer cell to independence [9]. To study the effect of AR overexpression, we stable transfected AR to LNCaP cell line leading to 3 to 4-fold increased expression of AR. The growth of LNCaP-ARhi was stimulated on lower concentrations of androgens than the growth of LNCaP-pcDNA3.1 (unpublished data). None of the coregulators were up- or downregulated by the level of AR alone since there was no significant difference in the expression of the coregulators at 0 M of DHT. Instead, the overexpression of AR seems to sensitize cells to androgens. This was evident *e.g. *for *PSA*, which was significantly upregulated more and in lower concentrations of DHT in LNCaP-ARhi than in LNCaP-pcDNA3.1 cells. Similarly, the expression of *MAK*, *CBP*, and *BRCA1 *was increased more in LNCaP-ARhi than in LNCaP-pcDNA3.1, and, at least, a 10-fold sensitization was observed. For *MAK *and *CBP *this was apparent in 4 h time point, whereas for *BRCA1 *at 24 h time point. Interestingly, the increased expression of AR did not affect all androgen-regulated coregulators. *β-catenin*, *cyclin D1 *and *gelsolin*, which showed androgen-regulation in LNCaP-pcDNA3.1 at 24 hours were not regulated by androgens in LNCaP-ARhi. The data suggest that the AR overexpression has distinct effects on different target genes. It should be recognized that during the androgen withdrawal, there are still adrenal androgens left. For example, the DHT concentration is only partially depleted [48]. Thus, the genes that are regulated in reduced DHT concentrations due to AR overexpression might be important in the progression of prostate cancer during the hormonal therapy.

The effects of androgens on gene expression were measured at two time points, 4 h and 24 h. The 4 h time points represent a time interval where direct effects of androgens administration should be seen, although the effect can be mild. On the other hand, also secondary mechanisms can already be activated at 24 hours time point. All coregulators (*AIB1*, *CBP*, *MAK*, *BRCA1*, *β-catenin*) that were induced more than 2-fold by DHT, showed the induction already at 4 time point in one or both cells lines (LNCaP-pcDNA3.1 and LNCaP-ARhi) suggesting direct regulation. Whereas, coregulators with lower-level of induction showed the effect variably in 4 and 24 hour points, which may also suggest secondary mechanisms of androgen regulation.

## Conclusion

In conclusion, by systematic measurement of 25 AR coregulators, we identified 5 coregulators whose expression seems to be significantly androgen-regulated indicating that they could be a part of positive feedback mechanisms potentiating the AR signaling. Overexpression of AR seems to sensitize cells in terms of androgen regulation but the effect is gene-specific since not all androgen-regulated genes were up-regulated in AR overexpressing model.

## Competing interests

The authors declare that they have no competing interests.

## Authors' contributions

AU: acquired the data through PCR, prepared the manuscript, analyzed and interpreted the data. KW: made the cell-culture work. HM: supported the PCR work. HS: established the AR overexpression cell lines. TV: designed the study, helped preparing the manuscript and analyzing and interpreting the data. All authors read and approved the final manuscript.

## Pre-publication history

The pre-publication history for this paper can be accessed here:



## Supplementary Material

Additional file 1 Additional Table 1 – primers' sequences and cycling conditions. primers' sequences and cycling conditions. Click here for file

Additional file 2 Additional Figure 1. Western blot analysis. Western analysis of β-catenin, CBP, and MAK in LNCaP-ARhi cells treated with different concentrations of DHT. Click here for file
