# Supplementary material for: Androgen regulation of the androgen receptor coregulators
Source: BMC Cancer. 2008 Aug 1;8:219. doi: 10.1186/1471-2407-8-219 (PMC2518564; doi:10.1186/1471-2407-8-219)
Supplement: Additional file 1 — Additional Table 1 – primers' sequences and cycling conditions. primers' sequences and cycling conditions. [file 1471-2407-8-219-S1.pdf]

Additional Table 1 - primers' sequences and cycling conditions.

Gene	Primer sequence (5'-3')	Annealing temperature (°C)	Temperature at the fluorescence measurement (°C)	Elongation time (sec)	Size of amplicon (bp)
PSA	GCAGCATTGAACCAGAGGAG<br>AGAACTGGGGAGGCTTGAGT	57	72	10	830
SRC1	ATGGTGAGCAGAGGCATGACA<br>AAACGGTGATGCTCATGTTG	60	72	14	349
TIF2	TAATGCACAGATGCTGGCC<br>TCTGTGTATGTGCCATTCGG	67	72	14	314
PIAS1	CCACATGACACCCATGCCTT<br>CCAAAGATGGATGCCGGGTC	67	72	14	333
PIASx[a]	TCTTCTGACGAAGAGGAAGACC<br>TCAGAAGATGTTCCAAGCTTCA	58	72	12	275
ARIP4	ATAGCAAGTTCCTACAGGGC<br>CAGATTCACACCCAAGCATC	61	72	18	437
BRCA1	TTCAGGGGGCTAGAAATCTG<br>CTACACTGTCCAACACCCACTCTC	62	72	10	247
β-catenin	AATACCATTCCATTGTTTGTGCAG<br>AGCTCAACTGAAAGCCGTTT	62	72	10	254
AIB3	TCCAGAACTTCTACCCAGCA<br>ATCAAGTCGCAGTCCTGCTT	61	72	14	344
AIB1	CGTCCTCCATATAACCGAGC<br>TCATAGGTTCCATTCTGCCG	54	72	10	255
CBP	CAGAGCGGATCATCCATGACTA<br>GCTTCTCCATGGTGGCATAC	59	83	14	347
STAT1	TATAGAGCATGAAATCAAGAGCC<br>GGGCATTCTGGGTAAGTTCA	55	72	10	227
NCoR1	GGCCCTCTTCAGTCTCCTCT<br>GGCAGGTTTTTGACCTGCTA	61	72	15	364
AES	CGCGATTGACATGATGTTTC<br>CTCTCAATGGCTCCCAAGAC	61	82	15	364
Cyclin D1	CCCTCGGTGGGTCCTACTTCAA<br>TGGCATTTTGGAGAGGAAGT	55	81	15	390
p300	CCTGAGTAGGGGCAACAAGA<br>GTGTCTCCACATGGTGCTTG	57	84	14	353
ARA24	CCACCAGAAGTTGTCATGGAC<br>ACAAGGGATGAGTTCACTTGC	57	82	11	475
LSD1	GCCTTCTGCTAAAGCCACAG<br>CGCCCTATGCAAGGAATATG	58	72	12	280
BAG-1L	GCTGACCAGCTGGAAGAGTT<br>TCTGGAAGGATCAGTGTGTC	60	72	14	167
Gelsolin	GATCGAAGAGGTTCCTGGTG<br>CCGCTTAGCAGAAGTCAAGG	59	72	9	148
PAK 6	ACATCGTCTCCCAAGTCAGG<br>GCCCAGAGACCAGATATCCA	58	72	15	293
MAK	TCCAAGATGAACCGATACACAACC<br>CAGCAATTTTCACAAGCTCTGGAC	59	72	19	433
Prohibitin	CGTGGGTACAGAAACCAATTATCT<br>CTCTCTCTGGGTGATTAGTTCTCC	61	72	13	257
JMJD1A	GCCAACATTGGAGACCACTT<br>CACCCTGTTGGCAATTCTTT	60	72	12	230
JMJD2C	GCTTGCGAGAAGGTCATTTC<br>GACTTCTCCCTCAGCAGGTG	58	72	10	207
MAGE11[b]	GGAGACTCAGTTCCGCAGAG<br>TGGGACCACTGTAGTTGTGG	59	72	10	170

a. Primers amplify a region that is common to the *PIASxα* and *PIASxβ* isoform
b. Primers amplify only a region of *MAGE11* transcript variant 1
