# Supplementary material for: Androgen regulation of the androgen receptor coregulators
Source: BMC Cancer. 2008 Aug 1;8:219. doi: 10.1186/1471-2407-8-219 (PMC2518564; doi:10.1186/1471-2407-8-219)
Supplement: Additional file 2 — Additional Figure 1. Western blot analysis. Western analysis of β-catenin, CBP, and MAK in LNCaP-ARhi cells treated with different concentrations of DHT. [file 1471-2407-8-219-S2.pdf]

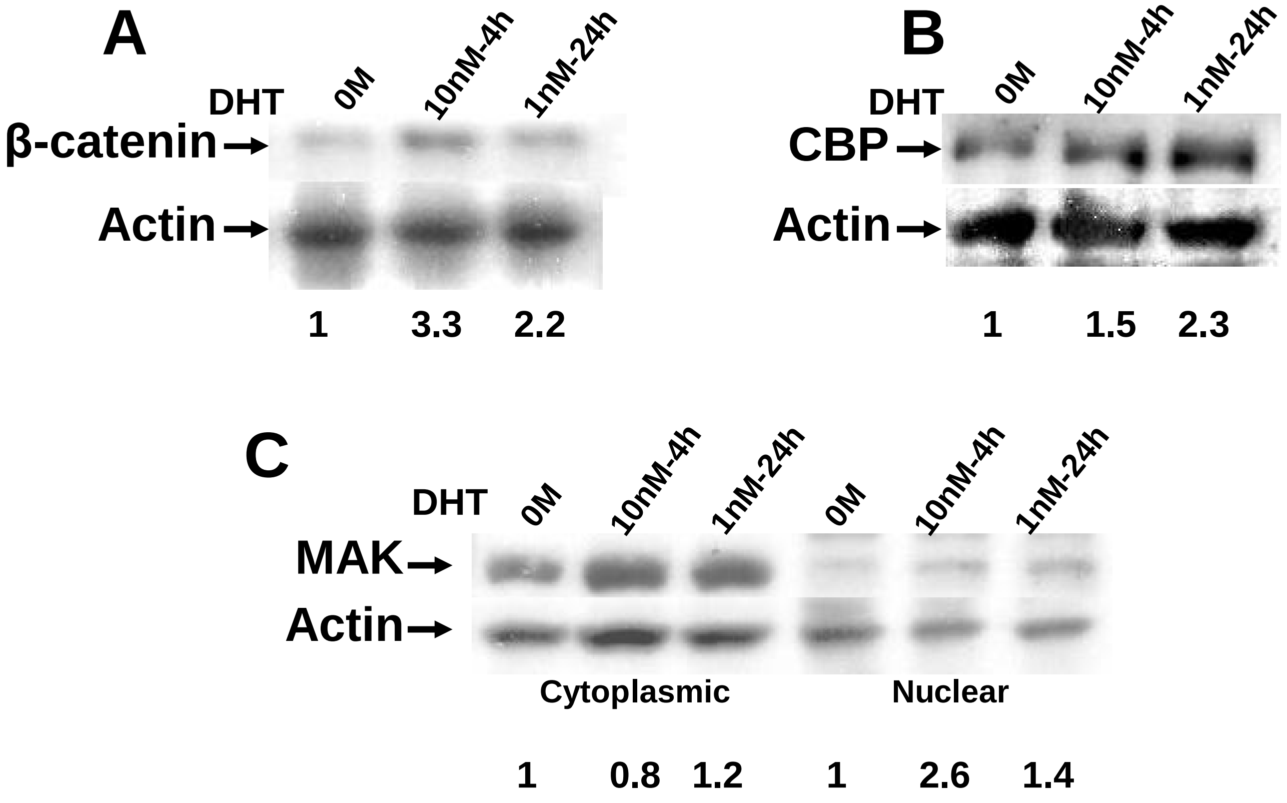

Additional Figure 1. Western blot analyses of nuclear A) β-catenin, and B) CBP, as well as cytoplasmic and nuclear C) MAK in LNCaP-ARhi. The ratiovalues of the protein of interest to pan-actin is given below each figures. The ratiovalues are normalized against the 0 M DHT treated cells. The analyses was repeated twice.
